# Perceived Gaze Direction Modulates Neural Processing of Prosocial Decision Making

**DOI:** 10.3389/fnhum.2018.00052

**Published:** 2018-02-13

**Authors:** Delin Sun, Robin Shao, Zhaoxin Wang, Tatia M. C. Lee

**Affiliations:** ^1^Laboratory of Neuropsychology, The University of Hong Kong, Pokfulam, Hong Kong; ^2^Laboratory of Cognitive Affective Neuroscience, The University of Hong Kong, Pokfulam, Hong Kong; ^3^Duke-UNC Brain Imaging and Analysis Center, Duke University, Durham, NC, United States; ^4^VA Mid-Atlantic Mental Illness Research, Education and Clinical Center (MIRECC), Durham, NC, United States; ^5^Shanghai Key Laboratory of Brain Functional Genomics, Key Laboratory of Brain Functional Genomics, Ministry of Education, Institute of Cognitive Neuroscience, School of Psychology and Cognitive Science, East China Normal University, Shanghai, China; ^6^The State Key Laboratory of Brain and Cognitive Sciences, The University of Hong Kong, Pokfulam, Hong Kong; ^7^Institute of Clinical Neuropsychology, The University of Hong Kong, Pokfulam, Hong Kong

**Keywords:** social decision making, eye gaze, fMRI, superior temporal gyrus, posterior cingulate cortex

## Abstract

Gaze direction is a common social cue implying potential interpersonal interaction. However, little is known about the neural processing of social decision making influenced by perceived gaze direction. Here, we employed functional magnetic resonance imaging (fMRI) method to investigate 27 females when they were engaging in an economic exchange game task during which photos of direct or averted eye gaze were shown. We found that, when averted but not direct gaze was presented, prosocial vs. selfish choices were associated with stronger activations in the right superior temporal gyrus (STG) as well as larger functional couplings between right STG and the posterior cingulate cortex (PCC). Moreover, stronger activations in right STG was associated with quicker actions for making prosocial choice accompanied with averted gaze. The findings suggest that, when the cue implying social contact is absent, the processing of understanding others’ intention and the relationship between self and others is more involved for making prosocial than selfish decisions. These findings could advance our understanding of the roles of subtle cues in influencing prosocial decision making, as well as shedding lights on deficient social cue processing and functioning among individuals with autism spectrum disorder (ASD).

## Introduction

Prosocial behaviors are the cornerstone of a harmonic society (Keltner et al., 2014), and are associated with complex considerations of benefits and intentions of both self and others (Rilling and Sanfey, 2011). Recent work showed that prosocial actions can be promoted in the presence of eyes or eye-like stimuli (Haley and Fessler, 2005; Nettle et al., 2013), suggesting the role of the eyes in effectively biasing social decision making. Gaze is one of the most important conduits of information delivered by eyes, and plays significant roles in social interaction (Itier and Batty, 2009; Carlin and Calder, 2013). However, little is known about the neural processing of social decision making modulated by perceived gaze direction.

Compared to selfish actions, prosocial decisions are more associated with attributions of the intentions and desires of counterparts, the so-called Theory of Mind (ToM; Baron-Cohen et al., 1985). According to this theory, understanding others’ needs and thoughts may promote the engagement in prosocial actions (Dunfield, 2014), while benefiting others may in turn contribute to the development of better ToM (Weller and Lagattuta, 2014). Consistent with this, a recent meta-analysis showed that ToM and prosocial behaviors are positively related in children (Imuta et al., 2016). Evidence also suggests the ability of paying attention to or understanding the information delivered by gaze appears in very early stage of development (Farroni et al., 2002). Someone else’s gaze informs us about the object or place he/she is looking at, and in turn how important or interesting such information is to him/her (Baron-Cohen, 1995; Shimojo et al., 2003; Frischen et al., 2007). Gaze direction has thus been proposed as a privileged stimulus for the attribution of mental state of others (Baron-Cohen, 1995). Direct gaze, usually accompanied with eye contact, indicates that someone is paying attention to us, while averted gaze implies the person is interested in people or objects other than us. On the other hand, Adams and Kleck (2003, 2005) found that direct gaze facilitates the processing of facial expressions indicating approach-oriented emotions (e.g., anger and joy), whereas averted gaze facilitates the processing of expressions implying avoidance-oriented emotions (e.g., fear and sadness). They thus proposed the “Shared Signal Hypothesis”, which postulates that the perception of a specific emotion will be enhanced when gaze direction matches the underlying behavioral intent communicated by that emotion expression. Taken together, direct gaze implies potential social contact and enhances the perception of approach-oriented emotions while averted gaze does not, which could in turn influence how readily we process others’ intentions and our subsequent social decision-making processes. However, no research has directly tested the effect of eye gaze direction on social decision making.

Brain regions related with ToM include the superior temporal gyrus (STG), posterior cingulate cortex (PCC), medial prefrontal cortex (mPFC), temporal-parietal junction (TPJ) and amygdala (Northoff and Bermpohl, 2004; Shaw et al., 2004; Schurz et al., 2014). These areas are widely implicated in social cognitive and decision-making processes (Moll et al., 2005, 2007; Rilling and Sanfey, 2011; Bastin et al., 2016). For example, Rilling et al. (2004) detected stronger activations in mPFC, PCC and TPJ when participants inferred the intent of human counterparts through their feedbacks during economic game tasks. Also, Moll and de Oliveira-Souza (2007) employed written statements describing action scenarios and found that prosocial emotions including guilt, embarrassment and compassion activated mPFC and bilateral STG. In a recent study, Morey et al. (2012) found that brief hypothetical scenarios in which the participants’ actions lead to harmful consequences to others vs. to self were associated with more intense feelings of guilt as well as stronger activations in mPFC, right STG and PCC. Further, amygdala was reported to signal the interaction between gaze direction and perceived facial expression (N’Diaye et al., 2009; Cristinzio et al., 2010; Sato et al., 2010; Ziaei et al., 2016, 2017), suggesting its role in the appraisal of self-relevance. These findings disclosed a positive relationship between prosocial actions/emotions and activations in the ToM brain network, and supported the idea that prosocial behaviors are related with more considerations of others’ thoughts and relationships between self and others.

Here, we were particularly interested in the STG. Existing imaging evidence indicates the right STG as being sensitive to gaze direction (Nummenmaa and Calder, 2009), suggesting its role as an eye direction detector (Baron-Cohen, 1995). Consistent with this, a patient with damage to the right STG showed difficulties in gaze discrimination and gaze-cued attention orientation (Akiyama et al., 2006). Stronger brain activations in right STG were also observed for direct than averted gaze (Calder et al., 2002; Pelphrey et al., 2004; but see Hardee et al., 2008). Previous studies have also found increased activations in the audience’s right STG when an actor tried to deceive the audience about the weight of a box he was lifting (Grezes et al., 2004a), when an actor had a false belief about the weight of the box (Grezes et al., 2004b), and when an actor chose an object he did not like or rejected an object he preferred (Wyk et al., 2009), suggesting that the STG is involved in detecting other’s intentions. Moreover, the STG may collaborate with other ToM areas to process gaze and social information, as it has both anatomical (Parvizi et al., 2006) and functional (Uddin et al., 2009) connections with the PCC. Stronger activations in bilateral STG, mPFC and PCC were reported in healthy participants when viewing direct than averted gaze (von dem Hagen et al., 2014), and greater mPFC-right STG functional connectivity was reported during social emotion, such as embarrassment and guilt, than basic emotion (Burnett and Blakemore, 2009).

In this study, we employed the functional magnetic resonance imaging (fMRI) method to investigate brain responses when participants were making either prosocial or selfish choices against anonymous counterparts in a novel economic exchanging game task (Sun et al., 2016). The effect of eye gaze direction was investigated by showing participants photos of counterparts’ eyes with either direct or averted gaze. In line with existing findings, prosocial vs. selfish choices were proposed to be associated with more considerations of others’ mental states as well as the relationship between self and others. In this context, prosocial choice was defined as behaviors that prioritize the benefit of other social, but not nonsocial (e.g., a robot), agents. We tested two types of relations between perceived gaze direction, social behaviors and the associated neural patterns. If prosocial choices were triggered by direct gaze that cues others’ intentions of social contact, we hypothesized to find stronger activations in right STG and larger functional connectivity between right STG and other ToM areas during making prosocial vs. selfish choices when perceiving direct than averted gaze. On the other hand, averted gaze signals the lack of intentions for social contact, and the viewers may need to make more cognitive efforts to infer the counterpart’s thoughts and to consider the relationship between self and others during making prosocial decisions. We then alternatively hypothesized to find stronger activations in right STG and larger functional connectivity between right STG and other ToM areas during making prosocial vs. selfish choices when detecting averted than direct gaze.

## Materials and Methods

### Participants

Thirty Chinese female university students (age = 24 ± 2.4 years, range = 20–29 years) participated in this study. Only females were recruited to avoid confounding gender influence in social decision making (Lee et al., 2009; Zhang et al., 2012; Sun et al., 2015b, 2016). All participants were right-handed (Oldfield, 1971) and had normal or corrected-to-normal vision. No participant had metal or medical device implants or any history of neurological or mental disorders. All participants in this study provided written informed consent to participate in procedures reviewed and approved by the local ethical committees at the University of Hong Kong and the East China Normal University. Three participants were excluded due to data recording errors, thus 27 participants were included for final analyses.

### Eye Stimuli

**Figure 1:**
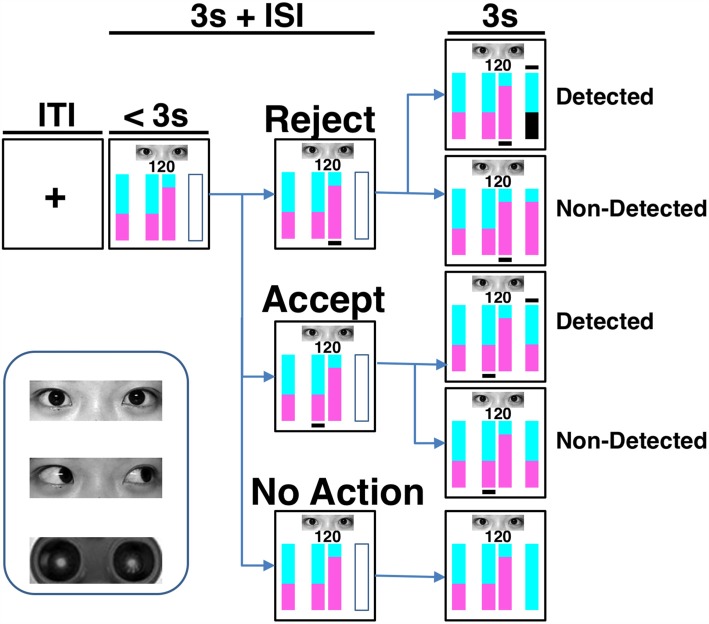
Task paradigm and eye stimuli. The cyan and purple areas in the vertically stacked bars represented the proportions of reward assigned to the counterpart and the participant, respectively. In each trial, after knowing the total amount to be divided (i.e., the number in the screen) and the counterpart’s offer (represented by the bar on the left-hand side), the participant could accept or reject the proposal by pressing one of two buttons corresponding to the bars in the middle of the screen within 3 s. To accept the counterpart’s proposal is beneficial to both players, while to reject the proposal indicates a plan more advantageous to the participant. Immediately after the choice action, a black line was shown beneath the corresponding bar. The final reward distribution of a trial was presented by the outcome bar on the right-hand side at the last 3 s of the trial. A black line appeared above the outcome bar if the real situation was detected. On the contrary, no line was shown if the detection did not occur. When a rejection was detected, the participant gained nothing in that trial and her area in the outcome bar became black. Under the other conditions, the participant kept her share. If there was no response, or should the response exceed the 3-s decision-making phase, the reward of the trial was sent to the counterpart. Both inter-trial-interval (ITI) and inter-stimulus-interval (ISI) were on average 3 s. One of the three cues was presented during each trial. The cues are human eyes with direct or averted gaze and robot eyes.

There were three types of eye photos: human eyes with a direct gaze, human eyes with an averted gaze and robot’s eyes (Figure 1). The photos of human eyes were collected from 24 volunteers (12 males and 12 females) prior to this study. Each volunteer gave two photos of his or her face with a front view and a neutral expression: one with a direct gaze and the other with an averted gaze (i.e., looking to the left). These photos were put into two sets, each containing six pictures of a male direct gaze, six pictures of a male averted gaze, six pictures of a female direct gaze, and six pictures of a female averted gaze. The two photos from the same volunteer never appeared in the same set to avoid the potential conflicts elicited by different gaze directions from the same volunteer. Half of the participants viewed the eyes in one set, while the other half viewed the other set of eyes. The photo of the robot’s eyes was modified from a cartoon robot’s eyes downloaded from Internet resources. All photos were of identical sizes and adapted to contain only the eye region before being changed into black and white through Adobe Photoshop software (San Jose, CA, USA).

### Task and Procedure

Each participant received the following instructions before experiment: “You are invited to interact with anonymous counterparts in an online game. You should treat each trial as a single-shot interaction since counterparts will vary across trials and players cannot recognize each other. A photo of either human eyes or a robot’s eyes will be shown on a given trial to represent the counterpart type, i.e., human or robot, but not identity. In each trial, you will receive from a counterpart both a monetary investment as well as an offer on how to divide between you two the final amount, which is the appreciated investment through a computer-mimicked stock market. For simplicity, you will be shown just the final amount but not the initial investment. You can accept the offer or reject it by choosing an alternative plan that is more beneficial to you but less advantageous to the counterpart. After that, the counterpart will have 50-50 chance to know whether you have accepted or rejected his/her offer. If you reject an offer, your share in that trial will be transferred to the counterpart when he/she knows your choice. In the other conditions, you will keep your share. If you make no action, all benefits will be delivered to the counterpart in that trial. Your choices will influence the actual incomes of players. That is to say, both you and the counterpart will get the corresponding amount if you accept the offer. However, if the human counterpart does not know that you have chosen an alternative option, you will gain more than offered and he/she less than offered. All human players but not robot will finally receive real monetary bonuses proportional to the amounts earned during the task”. Based on these instructions, the participant did not know that, in fact, computer programs mimicked all of the responses of human/robot counterparts. This approach was successful in our previous study utilizing similar task procedures (Sun et al., 2016). In order to reduce the influence of value calculation on participants’ choices, expected utility (i.e., reward × probability) on a given trial was equal between accepting and rejecting an offer.

In each trial (Figure 1), following a jittered inter-trial-interval (ITI) of 3 s (Poisson-distributed), the amount of the increased investment was shown on the screen for 3 s (decision phase) during which the participant had to make her choice by pressing one of two buttons with the right index or middle finger. During the decision phase, four vertical bars were displayed on the screen, with the leftmost bar reflecting the counterpart’s offer plan, the middle two bars reflecting the two available options for division to choose from, and the rightmost empty bar signaling the forthcoming outcome (yet to be revealed). One of the choice options corresponded to the counterpart’s proposal, whereas the other option gave the participant a greater potential monetary reward, but carried a 50% risk of gaining nothing. In the option bars, the proportions of reward assigned to the counterpart was represented by the cyan-colored area, and the reward assigned to the participant was represented by the purple-colored area (Figure 1). The number above the option bars indicated the total amount of appreciated investment to be divided. The spatial positions of the two choice options were randomized across trials. Once the participant had made a choice, a black line appeared underneath the selected option bar. After a jittered inter-stimulus interval (ISI) of 3 s (Poisson-distributed), the participant was notified whether the real situation was detected and how much she gained in that trial in the following 3 s (outcome phase). A black line above the outcome bar indicated that the counterpart knew the participant’s actual choice, while no such line was shown if the detection did not occur. When an action of rejecting the counterpart’s offer was detected, the participant gained nothing in that trial and her share in the outcome bar became black. Under the other conditions, the participant kept the share for herself. If the participant failed to make a choice in that trial, or if the response exceeded the 3-s interval, all reward would be sent to the counterpart.

There were a total of 144 trials in the formal task. Photos of a direct gaze, an averted gaze, and a robot’s eyes were each shown for 48 trials in randomized orders. The permutation of the offer—that is, the amount to be divided (a number randomly generated among 80, 100 and 150), the proposed portion of repayment to the counterpart (60%, 65%, 70%), and the location (left or right) of the bars representing two options were balanced across the different types of photos. Before getting into the scanner, each participant was given detailed instructions and completed a minimum of eight practice trials to ensure task comprehension. The photos used in the practice trials were different from those used in the formal task. All participants reported after the task that they believed they were playing with real human-being when the human eye stimuli were shown. The participants were debriefed after the experiment. Each participant was awarded 200 Chinese Yuan as compensation and also 0–100 Chinese Yuan (proportional to the task earnings) as a task bonus. The visual stimuli presentations and response collections were performed through the integrated functional imaging system (IFIS).

### Image Acquisition and Preprocessing

All images were acquired using a 3-Tesla Siemens Trio Tim MR scanner with a 12-channel head coil. T2*-weighted functional images were obtained using an EPI pulse sequence without inter-slice gap (33 axial slices parallel to the AC-PC line, TR = 2000 ms, TE = 30 ms, flip angle = 90°, Field of View (FOV) = 192 × 192 mm^2^, voxel size = 3 × 3 × 4 mm^3^). A high-resolution anatomical 3D T1-weighted MPRAGE image (192 slices, TR = 2530 ms, TE = 2.4 ms, flip angle = 7°, FOV = 224 × 256 mm^2^, voxel size = 0.5 × 0.5 × 1 mm^3^) was also acquired.

Images were preprocessed by using the CONN toolbox^1^, which calls functions from SPM12 software (Wellcome Department of Imaging Neuroscience, UK), through slice-timing and motion correction, normalization to the MNI (Montreal Neurological Institute) space and, finally, smoothing with an 8-mm full-width half-maximum Gaussian kernel.

### Statistical Analyses

Participants made actions (either accept or reject) in more than 94% trials. For our research aims, conditions involving interactions with robot served as a control conditions representing non-social-related processes such as general value-based decision making. We thus subtracted frequency of choice and mean reaction time (RT) related with robot counterpart from the corresponding data associated with human counterparts. No difference in choice frequencies was observed for human and robot counterpart trials (*p*s > 0.287). The four contrasts of interest were prosocial choice (i.e., accepting offer) accompanied with direct gaze, selfish choice (i.e., rejecting offer) accompanied with direct gaze, prosocial choice accompanied with averted gaze, and selfish choice accompanied with averted gaze. The frequency of choice and RTs were then respectively analyzed by a 2 (gaze direction: direct and averted) × 2 (choice: prosocial and selfish) repeated-measures ANOVA model.

Images were analyzed utilizing the SPM12 software. The general line model (GLM) was used to examine the experimental effects across task events within each participant. The onset of the decision phase was modeled by six regressors with 3-s duration which were combinations of eye stimuli (robot’s eyes, direct human eyes and averted human eyes) and action (accept and reject offers). In addition, one regressor modeled the choice response, one modeled the onset of the outcome phase (3-s duration), and six extra regressors modeling residual head motions were included as nuisances. These regressors were convolved with the SPM canonical hemodynamic response function. High-pass temporal filtering with a cut-off of 128 s was employed to remove low-frequency drifts.

To form within-subject contrasts, consistent with the approach of behavioral data analyses, beta-weight images of regressors of robot counterpart were subtracted from the corresponding images associated with human players. This approach gave four contrast images per participant, i.e., prosocial choice accompanied with direct gaze, selfish choice accompanied with direct gaze, prosocial choice accompanied with averted gaze, and selfish choice accompanied with averted gaze. These contrasts were then entered into a group-level 2 (gaze direction: direct and averted) × 2 (choice: prosocial and selfish) flexible factorial model. Results were voxel-level height thresholded at *p* < 0.001 and survived family-wise error (FWE) cluster-level correction (*p* < 0.05) within the whole brain. To specifically test our *a priori* hypotheses, we also reported findings with voxel-level height threshold at *p* < 0.001 and survived FWE correction (*p* < 0.05) within regions of interests (ROIs) including the PCC (Brodmann’s areas 23 and 31; Leech and Sharp, 2014), mPFC (Brodmann’s areas 9, 10, 24, 25 and 32; Murray et al., 2016), and bilateral amygdala constructed using the WFU_PickAtlas toolbox^2^). We also investigated the findings in bilateral anterior TPJ (center coordinates: left, *x* = −53, *y* = −30, *z* = 10; right, *x* = 47, *y* = −35, *z* = 12) and posterior TPJ (center coordinates: left, *x* = −53, *y* = −59, *z* = 20; right, *x* = 56, *y* = −56, *z* = 18) in spheres with a radius of 8 mm (Schurz et al., 2014). All significant clusters contained more than 5 voxels. Mean beta values of fMRI contrasts were extracted for further analyses from the aforementioned ROIs showing significant task activations, using the MarsBaR toolbox^3^. Bonferroni method was employed to correct for multiple comparisons during *post hoc* *t* tests and fMRI-behavior correlation analyses.

We further investigated the functional coupling between the seed region and the rest of the brain, especially in the ROIs of ToM areas. The seed area was the region showing significant interaction between gaze direction and choice in the fMRI analyses. We performed a generalized psychophysiological interaction (gPPI) analysis through the gPPI toolbox^4^. Following fMRI analyses, four contrast images per participant were made reflecting the differences between playing against human vs. robot. These contrasts were also entered into a group-level 2 (gaze direction: direct and averted) × 2 (choice: prosocial and selfish) flexible factorial model. Results were voxel-level height thresholded at *p* < 0.001, FWE cluster-level corrected at *p* < 0.05, and contained more than 5 voxels. Mean beta values of gPPI contrasts were extracted for further analyses from the significant cluster through the MarsBaR toolbox. Bonferroni method was employed to correct for multiple comparisons during *post hoc* *t* tests and behavior-fMRI correlation analyses.

## Results

### Behavioral Findings

Mean and standard deviations of behavioral measures were organized in Table 1. For our research purposes, behavioral data (i.e., frequency of choice and RT) and image contrasts related with robot counterpart were subtracted from the corresponding data associated with human counterparts. No significant gaze direction effects were found for either frequency of choice (*F*s < 2.292, *p*s > 0.142) or RT (*F*s < 0.858, *p*s > 0.363). Comparisons between human (including both direct and averted gaze conditions) and computer counterparts were organized in the supplementary document.

**Table 1:** Frequency of action and reaction time (RT).

Stimuli	Robot’s eyes		Human direct eyes		Human averted eyes	
Action	Accept	Reject	Accept	Reject	Accept	Reject
**Frequency (%)**
Mean	46.9	53.1	47.1	52.9	50.1	49.9
Std	13.5	13.5	12.9	12.9	12.1	12.1
**Reaction time (ms)**
Mean	1189.2	1143.1	1237.8	1217.9	1224.2	1223.4
Std	161.6	181.5	183.3	205.6	204.0	201.9

### fMRI Findings

**Figure 2:**
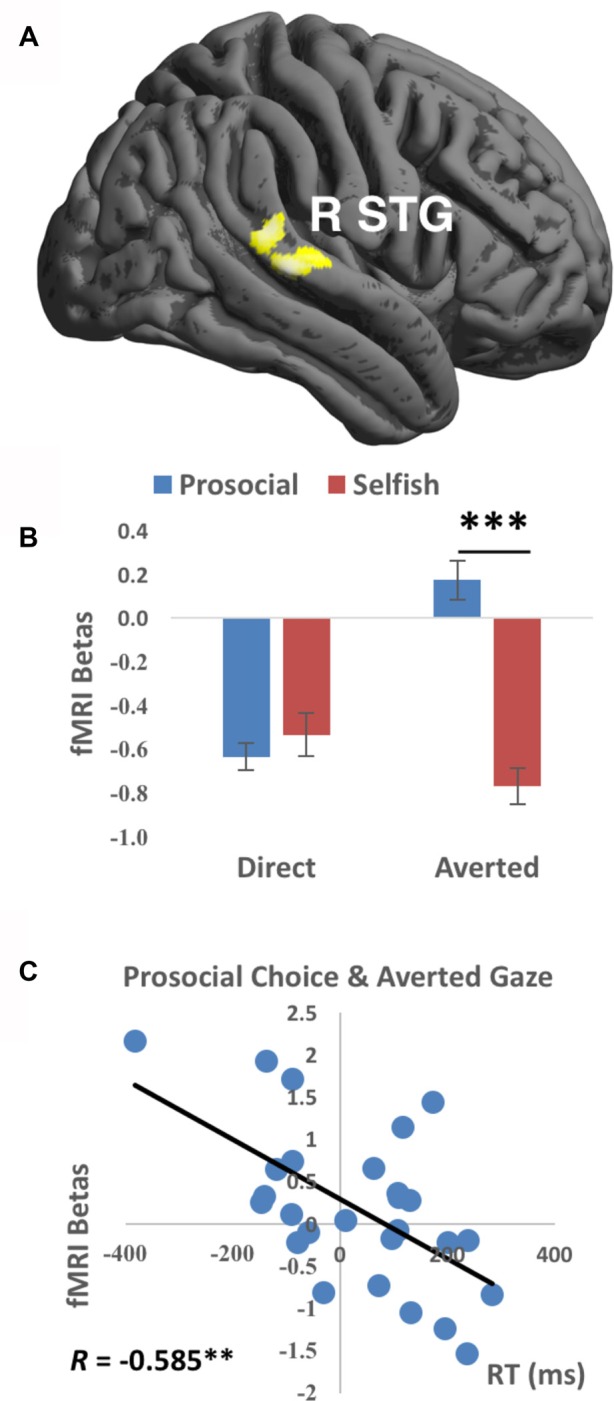
Functional magnetic resonance imaging (fMRI) findings of the interaction between Gaze and Choice. **(A)** Prosocial vs. selfish choices were associated with stronger activations in right superior temporal gyrus (R STG) for averted than direct gaze. Imaging results were height-thresholded at *p* < 0.001 and survived *p* < 0.05 family-wise error (FWE) correction. **(B)** Larger fMRI beta values averaged within the cluster in R STG were found for prosocial choice than selfish choice when averted gaze was presented (*t*_(26)_ = 4.583, *p* = 0.006 corrected). Error bar denotes standard error mean. **(C)** Significant correlation (Pearson’s *R* = −0.585, *p* = 0.008 corrected) was found between fMRI betas in R STG and reaction time (RT) for the condition of prosocial choice accompanied with averted gaze. **p < 0.01, ***p < 0.001.

Averted gaze elicited stronger activations in right STG (Brodmann’s area 22, cluster size = 293 voxels, T value = 4.18, peak MNI coordinates = [52, −46, 8], FWE-corrected cluster-level *p* value = 0.018) than direct gaze, while there was no significantly stronger activation for direct than averted gaze. No main effect of choice was detected significant. Importantly, a significant interaction between gaze direction and choice was detected in the right STG (Brodmann’s area 42/22, cluster size = 227 voxels, T value = 4.24, peak MNI coordinates = [70, −30, 16], FWE-corrected cluster-level *p* value = 0.047, see Figure 2A), characterized by stronger activations during prosocial choices vs. selfish choices when presented with averted gaze than direct gaze. Mean beta values extracted from this right STG cluster were greater to prosocial choices than to selfish choices when averted (*t*_(26)_ = 4.583, *p* < 0.001 corrected) but not direct (*t*_(26)_ = −0.459, *p* > 0.6) gaze was presented (Figure 2B). No significant results were found in the other ROIs. Comparisons between human (including both direct and averted gaze conditions) and computer counterparts were organized in the Supplementary Table S1. Moreover, larger mean betas in the right STG cluster were accompanied with quicker actions (i.e., shorter mean RT) across participants for prosocial choice to averted gaze (Pearson’s *R* = −0.585, *p* = 0.008 corrected, Figure 2C). No other correlation in right STG was found significant (all *p*s > 0.07 uncorrected), see Supplementary Table S2.

### gPPI Findings

**Figure 3:**
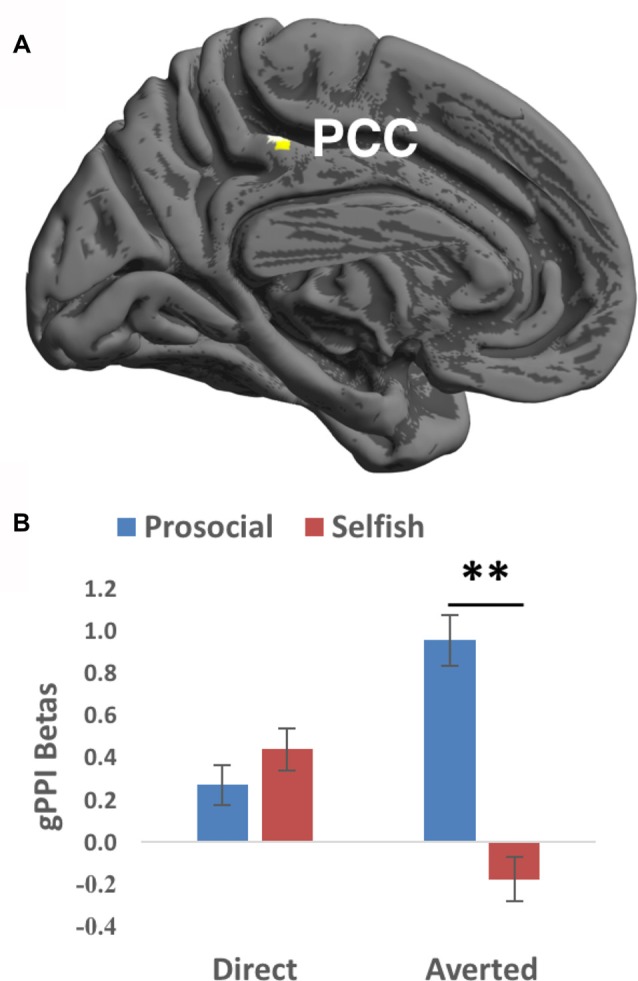
Generalized psychophysiological interaction (gPPI) findings of the interaction between Gaze and Choice. **(A)** Prosocial vs. selfish choices were associated with larger gPPI values in posterior cingulate cortex (PCC) for averted than direct gaze. Imaging results were height-thresholded at *p* < 0.001 and survived *p* < 0.05 FWE correction within an anatomical mask of PCC consisting of Brodmann’s area 23 and 31. **(B)** Larger gPPI beta values averaged within the cluster in PCC were found for prosocial choice than selfish choice when averted gaze was presented (*t*_(26)_ = 4.583, *p* = 0.006 corrected). Error bar denotes standard error mean. **p < 0.01.

We then investigated the functional couplings between the seed region and the rest of the brain, especially the ToM ROIs. The seed is the right STG cluster showing significant interaction between gaze direction and choice in the fMRI analyses. Prosocial choices vs. selfish choices were accompanied with larger gPPI values in PCC (surviving small-volume FWE correction; Brodmann’s area 31, cluster size = 26 voxels, T value = 4.29, Z value = 4.11, peak MNI coordinates = [−4, −34, 48], FWE-corrected peak-level *p* value = 0.009, Figure 3A) when averted gaze than direct gaze was shown. Mean beta values extracted from this PCC cluster showed that prosocial choices were accompanied with stronger functional couplings than selfish choices when averted (*t*_(26)_ = 3.703, *p* = 0.006 corrected) but not direct (*t*_(26)_ = −0.672, *p* > 0.5) gaze was presented (Figure 3B). No significant gPPI results were found in the other ROIs.

## Discussion

Eye gaze plays vital roles in many social contexts (Itier and Batty, 2009). However, little is known about its influences on social decision making integral to everyday social functioning (Rilling and Sanfey, 2011). To the best of our knowledge, this is the first study investigating the neural processing of the interaction between social decisions and perceived gaze direction. Consistent with the second *a priori* hypothesis, we found that prosocial vs. selfish choice elicited stronger activations in right STG and larger right STG-PCC functional connectivity when averted rather than direct gaze was presented. Moreover, stronger activations in right STG was associated with quicker actions for making prosocial choice accompanied with averted gaze. Our findings suggest that both right STG and right STG-PCC functional connections are more involved for making prosocial choices than selfish decisions when the perceived subtle social cues signal a lack of intentions for social contact.

The right STG has been widely reported to play roles in responding to gaze direction (Itier and Batty, 2009), and in detecting, predicting and reasoning about actions and intentions of others (Allison et al., 2000). Importantly, our findings further demonstrate the role of right STG in social decision making. We found stronger activation in right STG for prosocial than selfish choice accompanied with averted gaze. This result suggests that, when the perceived subtle cue through others’ gaze signals a lack of intentions for social contact, the right STG is more involved in inferring about the counterparts’ intentions during making a decision to benefit them. This explanation is further supported by the negative correlation between right STG activation and RT during prosocial choice accompanied with averted gaze. By contrast, no significant differences were found between prosocial and selfish choices accompanied with direct gaze. It is possible that being observed by others’ direct gaze is default in social interaction and carries a relatively constant level of processing about others’ intention regardless of the choice made.

Perception of observations by others has been found to efficiently promote prosocial behaviors (Izuma et al., 2010). It is hypothesized that, when being observed by someone, people make prosocial actions in order to gain social approvals/reputations (Rege and Telle, 2004; Izuma, 2012) and/or to avoid the guilt of harming others (Morey et al., 2012). Consistent with this idea, Izuma (2012) detected more donations to charities and stronger activations in striatum when donating in the presence of observers than in their absence. Direct (vs. averted) gaze is proposed to cue the observations by others and may thus elicit stronger brain activations for prosocial than selfish choices. However, this hypothesis is inconsistent with our findings. It is thus difficult to explain the gaze effect on social decision making through social reputation or guilt.

Previous studies have also detected stronger STG activations in response to the mismatch between one’s motion and the context (Grezes et al., 2004a,b; Wyk et al., 2009), and to moral judgments regarding the events that violate social norms (Prehn et al., 2008; Bahnemann et al., 2010). These findings suggest the roles of STG in reflecting the mismatch between observed actions and the context. If this theory also applies to the conflict between one’s own actions and the context, we would hypothesize to find stronger STG activations for both prosocial decisions accompanied with averted gaze and selfish choices accompanied with direct gaze. The reasoning is that prosocial decision is suboptimal when not being observed by others (represented by averted gaze implying higher chance of “getting away with” potential punishment), and selfish decision is suboptimal when being observed by others (represented by direct gaze implying high risk of being caught and punished). However, we only detected stronger brain activations for the former but not the latter condition, suggesting that our results cannot be fully interpreted by the conflict between one’s own actions and the context.

We also found larger functional couplings between right STG and PCC during making prosocial (vs. selfish) choices when perceiving averted gaze. The PCC has been widely reported in studies on self-referential processing (Lombardo et al., 2010; Brewer et al., 2013; Schurz et al., 2014). It is possible that more processing of others’ intentions (represented by stronger right STG activations) is accompanied with more processing of the relationship between self and others. This thought is consistent with the previous findings that the PCC as well as the nearby precuneus are associated with retrieval of episodic memory (Gobbini and Haxby, 2007; Sun et al., 2015c). In other words, during making prosocial decisions, when the perceived averted gaze implied lack of interpersonal interaction, participants need to recall their personal experiences in order to infer the others’ intentions.

We did not find any significant results in the other ToM ROIs including mPFC, TPJ and amygdala. First, mPFC has been proposed to play central roles in ToM (Schurz et al., 2014). However, Krause et al. (2012) utilized repetitive transcranial magnetic stimulation (rTMS) to bilateral mPFC, and did not find significant effect on either cognitive or affective ToM performance. They further reported that deep rTMS disrupted affective ToM performance in participants with high empathy, but increased affective ToM performance in those with low empathy, suggesting that the roles of mPFC in affective ToM are modulated by the baseline empathic abilities. In our study, the interaction between perceived gaze direction and social decision making may also be influenced by the level of empathy in participants which we unfortunately did not measure. Second, studies have shown that TPJ is sensitive to prediction error, which means the degree to which current information is inconsistent with expectation, in various domains including social interaction (Behrens et al., 2008; Simon et al., 2017). People in multiple-rounds social interactions may employ TPJ to guide behaviors based on previous experiences. However, participants in our task paradigm were instructed to treat each trial as a single-shot interaction, and they could not utilize the experiences with a previous counterpart to influence the current interaction. Third, amygdala has been widely reported to play a central role in response to the interaction between perceived gaze direction and facial expression (Cristinzio et al., 2010; Sato et al., 2010; Ziaei et al., 2016). Facial expression is delivered by not only eyes but also other parts of the face such as the mouth (Ekman, 2003; Sun et al., 2015a). In this study, however, the photos of humans were taken merely in neutral expression, and only the eye region was displayed. These might have minimized the roles of mPFC, TPJ and amygdala in our task paradigm. Future studies should further investigate the brain activation and functional connectivity in the ToM ROIs utilizing alternative task paradigms and stimuli, taking into account individual differences in social traits.

We failed to find any significant behavioral result related with either gaze or interaction between gaze and choice in this study. The presence of eye-like stimuli has been repeatedly reported to increases prosocial behaviors such as greater investments (Bente et al., 2014), greater charitable donations (Powell et al., 2012), theft prevention (Bateson et al., 2013) and higher voting rate (Panagopoulos, 2014), the so-called “watching eyes effect” (Nettle et al., 2013). There are two possible explanations for our insignificant findings. First, most of previous laboratory and field studies on the “watching eyes effect” employed procedures in which a participant makes decisions for only one or just a few trials (Haley and Fessler, 2005; Nettle et al., 2013). By contrast, participants in our task made choices in 144 trials. Second, the “watching eyes effect” were observed when eyes or eye-like stimuli were present vs. absent, while eye stimuli were always present in our study. The behavioral and neural mechanisms of gaze direction influence on social decision making may be different from those of the “watching eyes effect”. These need to be tested in future studies.

Our findings also have clinical implications, especially for people with autism spectrum disorders (ASDs) who exhibit impairments in reciprocal social interactions (Baron-Cohen et al., 1997). ASD has been found to relate to ToM impairments (Baron-Cohen et al., 2001). ASD patients show deficits in paying attention to the eye region (Spezio et al., 2007), extracting useful information from the eyes (Nation and Penny, 2008), and understanding others’ mental states (Campbell et al., 2006). A later diagnosis of autism was found to be predicted at 18 months of age by an absence of joint attention (Baron-Cohen et al., 1996), which is a precursor to ToM and reflects the ability of attending to the object cued by another person’s gaze direction (Emery, 2000). Our findings imply that ASD patients have deficits in processing subtle social cues in the environment such as eye gaze direction and/or in utilizing such information for making social decisions. Future work needs to clarify the neural underpinnings of social decision making in ASD patients when perceiving social cues. Further, previous studies suggested that brain stimulation techniques, such as TMS (Oberman et al., 2015) and Transcranial direct current stimulation (tDCS; Amatachaya et al., 2014, 2015), are promising methods of clinical treatment for ASD. These non-invasive brain stimulation techniques influence the activations in a small population of neurons in a targeted brain region. Our findings suggest that the right STG is a potential target for clinical intervention. Furthermore, the brain activation in the right STG and the right STG-PCC functional connections may also be employed to reflect the outputs of clinical treatment.

Our study have several limitations. First, our participants were all Chinese females. Caucasian and East Asian participants were found to fixate on the internal features (especially the eyes) and the center of faces (Blais et al., 2008), respectively, suggesting culture influences on face processing. People in western cultures may thus be more influenced by gaze direction while making social decisions. Future studies are needed for comparing the gaze-orienting effects across cultures and in different gender groups. Second, direct and averted gazes in this study were all shown in photos containing front-view faces, and were restricted within the eye region for simplicity. Previous studies have shown that head orientation (Itier et al., 2007) and dynamic gaze presentation (Putman et al., 2006) influenced the effects of gaze. Future studies should investigate the influence of gaze direction on social decision making in different head orientations and/or using a dynamic gaze (e.g., movies of gaze motion or real human eyes). Third, only left-oriented eyes were employed to represent the averted gaze in this study. Different directions of averted gaze may confer different social meanings and recruited different STG subregions in Calder et al. (2007). Future studies should separately investigate the effects of different averted gaze directions.

In conclusion, our study provides the first evidence that the neural processing of social decision making is influenced by perceived gaze direction. Our findings suggest that, when the perceived gaze direction signals lack of social contact, making prosocial vs. selfish decisions is associated with greater neural processing of inferring others’ intention and understanding the relationship between self and others. These findings also shed light on the deficiencies in processing subtle social cues and social functioning in ASD individuals.

## Author Contributions

DS, RS, ZW and TMCL made substantial contributions to the conception or design of the work or the acquisition, analysis, or interpretation of data for the work; drafted the work or revised it critically for important intellectual content; approved the final version to be published; agreed to be accountable for all aspects of the work in ensuring that questions related to the accuracy or integrity of any part of the work are appropriately investigated and resolved.

## Conflict of Interest Statement

The authors declare that the research was conducted in the absence of any commercial or financial relationships that could be construed as a potential conflict of interest.

## Footnotes

^1^https://sites.google.com/view/conn/

^2^http://fmri.wfubmc.edu/software/pickatlas

^3^http://marsbar.sourceforge.net/

^4^http://www.nitrc.org/projects/gppi
