# Supplementary material for: Perceived Gaze Direction Modulates Neural Processing of Prosocial Decision Making
Source: Front Hum Neurosci. 2018 Feb 13;12:52. doi: 10.3389/fnhum.2018.00052 (PMC5816754; doi:10.3389/fnhum.2018.00052)
Supplement: Supplementary file 1 [file Table_1.docx]

**Supplementary Document**

**Perceived Gaze Direction Modulates Neural Processing of Prosocial Decision Making**

Delin Sun ^1,2,3,4^, Robin Shao ^1,2^, Zhaoxin Wang^5^, Tatia M.C. Lee ^1,2,6,7,*^

^1^ Laboratory of Neuropsychology, The University of Hong Kong, Hong Kong.

^2^ Laboratory of Cognitive Affective Neuroscience, The University of Hong Kong, Hong Kong.

^3^ Duke-UNC Brain Imaging and Analysis Center, Duke University, Durham, NC, United States.

^4^ VA Mid-Atlantic Mental Illness Research, Education and Clinical Center (MIRECC), Durham, NC, United States.

^5^ Shanghai Key Laboratory of Brain Functional Genomics, Key Laboratory of Brain Functional Genomics, Ministry of Education, Institute of Cognitive Neuroscience, School of Psychology and Cognitive Science, East China Normal University, Shanghai, China.

^6^ The State Key Laboratory of Brain and Cognitive Sciences, The University of Hong Kong, Hong Kong.

^7^ Institute of Clinical Neuropsychology, The University of Hong Kong, Hong Kong.

* **Correspondence to:**

Tatia M.C. Lee, Ph.D.

May Professor in Neuropsychology

Rm 656, Jockey Club Tower

The University of Hong Kong

Pokfulam Road

Hong Kong

Tel: (852) 3917-8394

Email: tmclee@hku.hk

**Human versus Computer Counterpart**

We analyzed the behavioral and fMRI data to investigate the differences between human and computer counterpart, which reflect the differences between social and non-social decision making.

For the behavioral data analysis, we merged the data related with either human direct gaze or human averted gaze into one dataset, i.e. human eyes, for the choices of accept and reject, respectively. We then employed a 2 (Counterpart: human vs computer) by 2 (Choice: accept vs reject) repeated measures ANOVA model to analyse frequency of choice and reaction time data using the SPSS software (ver. 24, IBM Inc.). For frequency of choice, no significant result was found for Counterpart (*F*(1, 26) = 0.000, *p* = 1.000), Choice (*F*(1, 26) = 1.432, *p* = 0.242) and their interaction (*F*(1, 26) = 0.307, *p* = 0.584). On the other hand, for reaction time, we found significant effect of Counterpart (*F*(1, 26) = 11.234, *p* = 0.002), depicting that human counterparts (mean±SD: 1225.9±33.6 ms) were related with longer reaction time than the computer counterpart (1166.1±30.0 ms). No significant results were found for the effect of Choice (*F*(1, 26) = 1.866, *p* = 0.184) or the Counterpart X Choice interaction (*F*(1, 26) = 0.859, *p* = 0.363).

For the fMRI data analysis, we averaged the contrast images related with either human direct gaze or human averted gaze into one, i.e. human eyes, for the accept choice and reject choice, respectively. We then entered the contrast images from all subjects into a 2 (Counterpart: human vs computer) by 2 (Choice: accept vs reject) flexible factorial model using SPM. The statistical thresholds were consistent with those described in the main text. It is clear that the two factors and their interaction were related with significant brain activations in several brain areas. The results were listed in Table 1S.

Behavioral results showed that playing against human counterparts were associated with longer reaction time than against the computer counterpart. Moreover, the brain activation map was different between playing against the two types of counterparts. These findings suggest that different processing was employed between interacting with human and computer counterpart.

Table 1S.

|  |  |  |  | **MNI coordinates** | | |
| --- | --- | --- | --- | --- | --- | --- |
| **Area** | **k** | **T** | **Z** | **x** | **y** | **z** |
| ***Human > Computer Counterpart*** | | | | | | |
| L/R Occipital Cortex (BA17/18/19) | 19416 | 12.23 | Inf | -30 | -84 | 8 |
| R Middle Frontal Gyrus (BA9/46) | 1762 | 6.96 | 6.29 | 58 | 26 | 26 |
| L Insula (BA13) | 892 | 6.34 | 5.81 | -40 | -4 | -4 |
| L/R Supplementary Motor Area (BA6) | 569 | 5.80 | 5.39 | 4 | 0 | 76 |
| L Middle Frontal Gyrus (BA46) | 298 | 5.40 | 5.06 | -44 | 34 | 18 |
| R Insula (BA13) | 316 | 5.16 | 4.86 | 38 | 0 | 2 |
|  |  |  |  |  |  |  |
| ***Human < Computer Counterpart*** | | | | | | |
| L Angular Gyrus (BA39/40) | 1229 | 7.70 | 6.83 | -54 | -62 | 40 |
| R Angular Gyrus (BA39/40) | 548 | 6.83 | 6.20 | 52 | -66 | 38 |
| R Superior Temporal Gyrus (BA21/22) | 549 | 6.53 | 5.97 | 68 | -38 | 14 |
| L/R Thalamus | 856 | 6.21 | 5.72 | -8 | -18 | -4 |
| R Cuneus (BA18/19) | 488 | 5.87 | 5.44 | 10 | -84 | 30 |
| R Supplementary Motor Area (BA6) | 401 | 5.48 | 5.13 | 16 | -8 | 60 |
| R Insula (BA13) | 188 | 5.20 | 4.89 | 38 | -32 | 20 |
| L Middle Frontal Gyrus (BA8/9) | 322 | 4.56 | 4.34 | -28 | 28 | 38 |
| L Supplementary Motor Area (BA6) | 365 | 4.49 | 4.28 | -10 | 6 | 58 |
| L Posterior Cingulate Cortex (BA31) | 457 | 4.49 | 4.28 | -2 | -42 | 36 |
| L Anterior Cingulate Cortex (BA32/10) | 377 | 4.22 | 4.04 | -10 | 36 | 10 |
|  |  |  |  |  |  |  |
| ***Reject > Accept*** | | | | | | |
| R Superior Temporal Gyrus (BA22) | 733 | 7.15 | 6.43 | 68 | -38 | 16 |
| L Angular Gyrus (BA39/40) | 415 | 6.83 | 6.20 | -42 | -74 | 46 |
| L/R Thalamus | 1048 | 6.48 | 5.92 | -8 | -20 | -6 |
| L Posterior Cingulate Cortex (BA31/23) | 1667 | 6.13 | 5.65 | 8 | -52 | 18 |
| L Supplementary Motor Area (BA6) | 191 | 5.75 | 5.35 | -8 | 6 | 56 |
| L Middle Frontal Gyrus (BA9) | 368 | 5.44 | 5.09 | -26 | 24 | 38 |
| R Cuneus (BA18/19) | 538 | 4.98 | 4.71 | 10 | -86 | 30 |
| L/R Ventral Medial Prefrontal Gyrus (BA32/10/11) | 721 | 4.39 | 4.19 | 8 | 32 | -10 |
|  |  |  |  |  |  |  |
| ***Reject < Accept*** | | | | | | |
| L/R Occipital Cortex (BA17/18/19) | 17662 | 11.32 | Inf | -30 | -84 | 8 |
| L Hippocampus | 1536 | 8.19 | 7.18 | -22 | -30 | -6 |
| R Hippocampus | 271 | 7.09 | 6.39 | 22 | -30 | -4 |
| R Amygdala | 71 | 5.13 | 4.83 | 22 | -2 | -22 |
| L/R Supplementary Motor Area (BA6) | 437 | 4.96 | 4.69 | 4 | 2 | 74 |
| R Middle Frontal Gyrus (BA6/8/9/46) | 300 | 4.72 | 4.48 | 50 | 12 | 50 |
|  |  |  |  |  |  |  |
| ***(Reject-Accept)_Human_ > (Reject-Accept)_Computer_*** | | | | | | |
| R Superior Temporal Gyrus (BA22) | 152 | 5.48 | 5.13 | 68 | -36 | 14 |
| L/R Posterior Cingulate Cortex (BA30/23) | 152 | 5.07 | 4.78 | 8 | -52 | 18 |
| L Angular Gyrus (BA39/40) | 240 | 4.84 | 4.58 | -42 | -74 | 46 |
|  |  |  |  |  |  |  |
| ***(Reject-Accept)_Human_ < (Reject-Accept)_Computer_*** | | | | | | |
| L/R Occipital Cortex (BA17/18/19) | 26143 | 11.33 | Inf | -30 | -84 | 8 |
| R Middle Frontal Gyrus (BA6) | 320 | 5.44 | 5.09 | 50 | 12 | 50 |
| L Middle Frontal Gyrus (BA9/46/10) | 366 | 4.37 | 4.17 | -38 | 44 | 30 |

Note: results were height-thresholded at *p* < 0.001 and extension-thresholded at > 5 voxels, and survived *p* < 0.05 FWE correction within the whole brain. Area, name of brain areas; k, cluster size; T and Z, T and Z values; BA, Brodmann's area; L = left, R = right.

Table 2S. Correlations between fMRI betas in the right STG cluster and behavioral performance.

| **Condition** | **R** | **p uncorr.** | **p corr.** |
| --- | --- | --- | --- |
| ***Correlation with Frequency of Choice*** | | |  |
| Prosocial & Direct Gaze | 0.124 | 0.539 | 4.312 |
| Selfish & Direct Gaze | -0.253 | 0.202 | 1.616 |
| Prosocial & Averted Gaze | 0.233 | 0.241 | 1.928 |
| Selfish & Averted Gaze | -0.141 | 0.483 | 3.864 |
|  |  |  |  |
| ***Correlation with Reaction Time*** | | |  |
| Prosocial & Direct Gaze | -0.353 | 0.071 | 0.568 |
| Selfish & Direct Gaze | -0.308 | 0.118 | 0.944 |
| Prosocial & Averted Gaze | -0.585 | 0.001 | 0.008 |
| Selfish & Averted Gaze | -0.244 | 0.219 | 1.752 |

Note: R, Pearson's correlation coefficient; p uncorr., uncorrected p values; p corr., p values corrected by Bonferroni method.
